# Blockchain Enabled Transparent and Anti-Counterfeiting Supply of COVID-19 Vaccine Vials

**DOI:** 10.3390/vaccines9111239

**Published:** 2021-10-25

**Authors:** Harsha Chauhan, Deepali Gupta, Sheifali Gupta, Aman Singh, Hani Moaiteq Aljahdali, Nitin Goyal, Irene Delgado Noya, Seifedine Kadry

**Affiliations:** 1 Chitkara University Institute of Engineering & Technology, Chitkara University, Rajpura 140401, India; harsha.chauhan@chitkara.edu.in (H.C.); deepali.gupta@chitkara.edu.in (D.G.); sheifali.gupta@chitkara.edu.in (S.G.); 2 Department of Computer Science and Engineering, Lovely Professional University, Jalandhar 144001, India; aman.16826@lpu.co.in; 3 Faculty of Computing and Information Technology, King Abdulaziz University, Jeddah 37848, Saudi Arabia; Hmaljahdali@kau.edu.sa; 4 Higher Polytechnic School, Universidad Europea del Atlántico, C/Isabel Torres 21, 39011 Santander, Spain; irene.delgado@uneatlantico.es; 5 Department of Project Management, Universidad Internacional Iberoamericana, Campeche 24560, Mexico; 6 Faculty of Applied Computing and Technology, Noroff University College, 0459 Kristiansand, Norway; seifedine.kadry@noroff.no

**Keywords:** blockchain, COVID-19, vaccine supply, transparency, Ethereum, smart contract

## Abstract

The COVID-19 pandemic has profoundly affected almost all facets of peoples’ lives, various economic areas and regions of the world. In such a situation implementation of a vaccination can be viewed as essential but its success will be dependent on availability and transparency in the distribution process that will be shared among the stakeholders. Various distributed ledgers (DLTs) such as blockchain provide an open, public, immutable system that has numerous applications due the mentioned abilities. In this paper the authors have proposed a solution based on blockchain to increase the security and transparency in the tracing of COVID-19 vaccination vials. Smart contracts have been developed to monitor the supply, distribution of vaccination vials. The proposed solution will help to generate a tamper-proof and secure environment for the distribution of COVID-19 vaccination vials. Proof of delivery is used as a consensus mechanism for the proposed solution. A feedback feature is also implemented in order to track the vials lot in case of any side effect cause to the patient. The authors have implemented and tested the proposed solution using Ethereum test network, RinkeyBy, MetaMask, one clicks DApp. The proposed solution shows promising results in terms of throughput and scalability.

## 1. Introduction

The World Health Organization (WHO) declared a global pandemic due to COVID-19 on 30 January 2020 [1]. Health and economic losses, due to the pandemic in addition to sanitization and social distancing has dragged the world into a highly arduous situation. COVID-19 belongs to the coronavirus ribonucleic acid virus family that can easily spread from person to person [2]. After the identification of COVID-19 in Wuhan, China in December 2019 it spread swiftly as it is a communicable disease generating up to June 2021 around 181,722,790 confirmed positive cases and 3,942,233 deaths worldwide [3,4]. despite significant actions such as lock-downs undertaken by governments to fight and to slow down the transmission rate of the COVID-19 virus. In many countries, protocols and restrictions are even now still in place to prevent the collapse of various COVID healthcare centers [5].

In order to control the effect of COVID-19 the initiation and implementation of the immunity a vaccine campaign is important. From the start of the COVID-19 pandemic, many pharmaceutical companies Pfizer, Covaxin, Covisheild, etc. have deployed vaccines in market to achieve the containment of COVID-19 [6,7]. However, many aspects are prone to affect the successful implementation of the immunization program of COVID-19 such as counterfeiting, vial security and distribution issues, patient registration data security issues, tempering with data. [8]. In order to address the technical challenges blockchain can be a solution.

Almost all the immunization vaccines are developed using the same layer of protein that is present on the surface of coronavirus. The role of these vaccines is to make the immune system of human beings familiar with the virus, so that when virus will attach it will be able to reorganize [9]. The m-RNA vaccination produces the same contaminated protein of the virus in human cells. Though the downside with such immunity vaccines is that they become ineffective and useless if not stored at the recommended temperature [10]. The primary aspect of aforementioned limitation is the transparency available in end-to-end supply chain management and logistics management [11,12]. The role of this end-to-end supply chain is to ensure and keep the track of temperature, also control in the cold chain during storage and distribution of immunization vaccines which is a universal priority. Table 1 presents the parameters of the various different immunization vaccines during storage and distribution.

Distributed ledgers (DTLs) such as blockchain can enhance the transparency and efficiency of the distribution of COVID-19 vaccine. Blockchain stores the data in an immutable environment and thus assures the traceability of storage and delivery conditions. A solution based on blockchain technology may cater to completely self-acting implementation of information liability and data provenance track during distribution of COVID-19 vaccine. Hence, it will provide the amalgamation with various data divisions that are managed by different nodes on an entirely decentralized distributed chain. The self-regulating smart-contracts can ensure the supply traceability in the supply chain of COVID-19 vaccines. During the distribution and storage of vaccines it is important to keep a track of temperature to remain viable. The peer of the network will be able to keep track of the data as it is stored in a distributed ledger. Finally, DTLs data sure as a proof-of-delivery (POD) chain that will make it complex and difficult to counterfeit the vaccine. With the help of blockchain technology the medical units and vaccine stakeholders can check the details related to the vaccine.

In this article, the authors have proposed a blockchain-enabled framework for transparent supply chain management of COVID-19 vaccine. The framework provides a transparent track of registration, delivery and self-reporting. Leveraging blockchain will introduce the following features of the proposed framework:

- DLTs-based framework for immutable data, transparency and efficiency of registration process for COVID-19 vaccination in order to avoid counterfeit and identification theft.
- Smart contract enabled framework for self-administering the vaccine distribution constraints in the cold chain about the fulfilment of COVID-19 vaccine. The framework for vaccine supply chain management will enable the features of tamper-proof, person identification and avoidance of counterfeiting.
- The proposed solution helps vaccination centers to monitor the process of vaccination; it will also help patients register in a secure manner.

The organization of remaining paper is as follows: In Section 2 the related literature reviews in the area of blockchain, immunization vaccines and supply chain management will be discussed. Section 3 includes the method and procedure of the proposed framework. The environmental setup and results have been discussed in Section 4. Finally, Section 5 presents the conclusions and future scope.

## 2. Related Work

Communication and information technology-based vaccination schemes are presented in the related work for optimal supply and distribution of COVID-19 vaccines [16,17,18,19,20,21]. A drive through simulation for mass vaccination in a feasible manner during COVID-19 pandemic has been proposed that provides assessment of such facilities such as agent-based modelling and event processing to reduce the waiting times, required staff, intervals of immunization, etc. [22]. Supply and distribution of COVID-19 vaccines for heterogeneous populations is done by using computational equity and modelling constraints in order to maintain the optimization and fairness in case of an infection outbreak [23]. Many custom and heuristic algorithms have been proposed to optimize the design and flow of distributed networks [24,25,26,27].

Latest furtherance of emerging technologies, especially the internet-of-things (IoT), artificial intelligence (AI) and blockchain offers a path for developing more innovative and smart systems that might be used in various areas such as in the healthcare domain [28]. A solution for ensuring the transparency and increasing the coverage of vaccines in remote regions is using IoT sensor devices to track the carrier’s location, humidity and temperature [29]. Blockchain-enabled solutions addressing the challenges such as confidentiality, security and privacy of the data are described in [30]. In recent studies the possibility of combating the COVID-19 pandemic with blockchain solutions to provide decentralized tracking of contracts and enabling features such as data safety and immutability has been pointed out [31,32]. Use cases related to technology like blockchain can help to manage the pandemic of COVID-19 by tracing contacts, data sharing of patients, supply and distribution management are presented in [33]. Blockchain technology may be used to establish trustworthy prediction models that will help in handling the COVID-19 pandemic crisis on national level [34]. Also, to track the location during quarantine places in a secure manner using IoT sensor node infrastructure [35]. A blockchain-enabled incentive-based approach was proposed to fight against the pandemic to avoid information tampering and grant incentive tokens to patients for completing their quarantine time [36].

Blockchain has provided enormous solutions for the management and organization of supply chain management [37]. Various integrated frameworks of blockchain and IoT for the supply chain of pharmaceuticals where the counterfeiting prevention and temperature monitoring of drugs are of utmost importance [38]. A recommendation system using blockchain and machine learning for the management of the drugs supply chain has been proposed [39]. Hyperledger fabrics used for the deployment that continuously audit and make a track of the delivery process while LightGBM and N-grams used to recommend the best drugs to the clients. A solution for transparent drug data flow using the Gcoin blockchain in order to eliminate the counterfeiting of drugs has been given [40].

Few developed solutions in the presented state of art have provided a solution based on blockchain for the distribution of vaccines. A management system based on blockchain to supervise the supply chain of vaccines through smart contracts, also the system manages the vaccine expiration and fraud records has been proposed [41]. A blockchain framework for the of COVID-19 vaccine has given. The solution proposes administering of each process from the development to the commercial chain by leveraging the blockchain for registering charges and authentication of each process [42]. Likewise, a solution for a proficient supply chain using IoT and smart contracts has been proposed, the system consists of various IoT sensors and used to monitor legitimize receivers, shipment etc. [43]. A blockchain enabled framework for administer the production and tracing of vaccines using IoT sensors in China has been proposed. The device captures vaccine data during production and stores the data on the blockchain network in order to maintain the immutability [44]. Additionally, blockchain can be used to track the vaccines and have not been compromised or even to maintain the track of vaccine recipients especially because COVID-19 vaccine process requires two doses for a successful campaign [45,46].

## 3. Methodology

Immunization vials contain fragile biological substances, and if exposed to temperatures outside the recommended limit or opened in a non-suitable environment vaccines can lose their effectiveness and efficacy. The processes of storing, distributing and monitoring vials are difficult to manage and need cautions while handling and as to beneficiary’s privacy, it is the most critical point. In this paper, the authors have proposed a blockchain-based solution to help and automate the supply of COVID-19 vaccines. In the following subsections the authors have given system architecture, system workflow and system algorithms.

### 3.1. System Architecture

**Figure 1:**
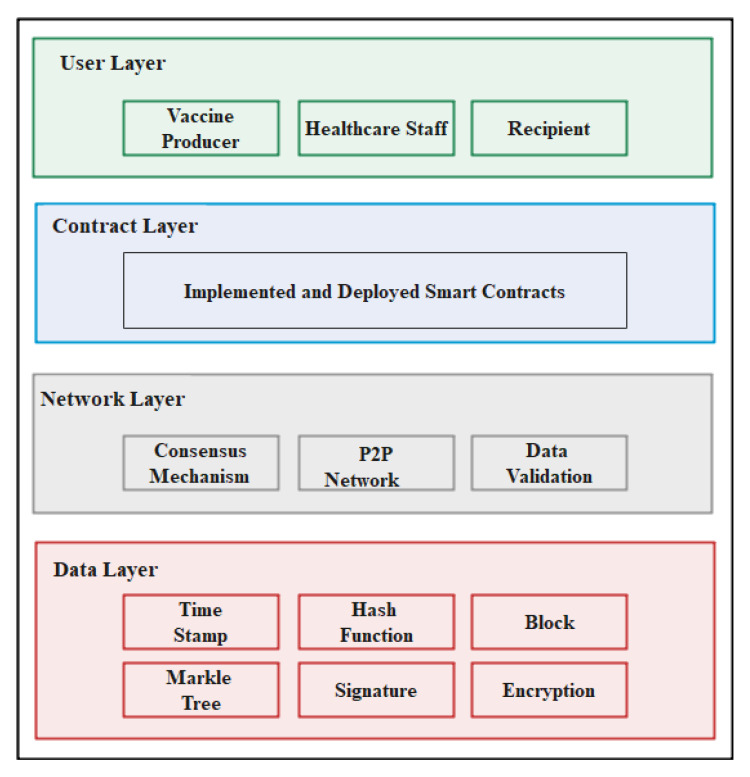
System architecture of the proposed solution.

The system architecture of the proposed solution consists of four layers: user layer, smart contract layer, network layer and data layer as shown in Figure 1. 

The data layer and network layer are part of the blockchain environment whereby the data layer acts as a core layer that is responsible for the storing and chaining of data whereas the network layer’s task is to perform transmission of data, data validation, consensus mechanisms and peer-to-peer networking in the blockchain network. The smart contract layer contains all the contracts that are responsible for every event done in the network and the smart contracts layer also acts as the core of the complete system that encapsulate the coding and delivers the functions to the system [47]. Smart contracts include the code and deployment for vaccine distribution tractability and anti-counterfeiting. The user layer, also called the application layer, allows the recipient, healthcare workers and all the peers in the vaccine distribution system to interact with the interface of the blockchain network.

### 3.2. System Work Flow

**Figure 2:**
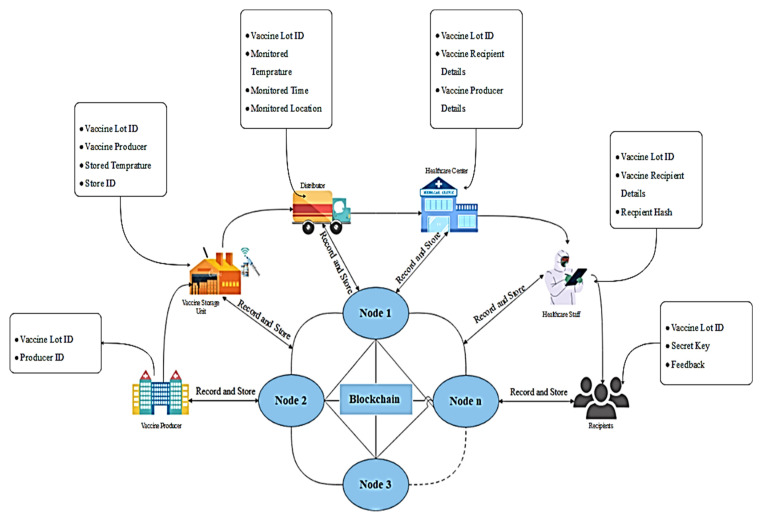
Architecture of the proposed framework for transparent supply of COVID-19 vaccines.

The architecture of the proposed blockchain-enabled solution for transparent supply of COVID-19 vaccine is presented in Figure 2. The entire track data related to COVID-19 vaccine during supply is stored in distributed ledgers over blockchain networks in order to assure the immutability of data and guarantees that the COVID-19 vaccines vials are transported safely.

The main participants of the proposed framework considered as the peer nodes of blockchain network are: (i) the recipient who will register for immunization, (ii) the laboratory or pharmaceutical factories that will prepare the vials of vaccine, (iii) the IoT sensors which will monitor the temperature and location at the time of distribution, (iv) the healthcare centers which will provide the vaccine services to the vaccine recipients, (v) the healthcare workers who will verify the details of recipient during the vaccination process also they will control the vaccine conditions during delivery and storage.

**Figure 3:**
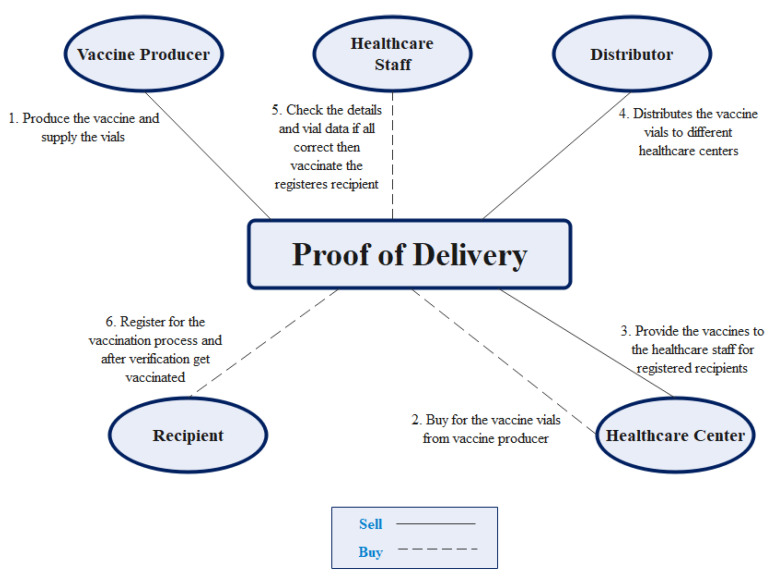
Proof of delivery.

All the data of all the actions is stored in distributed ledgers that are immutable in nature and is accessible by all the participants of the network. Thus, it enables the feature of transparency at each level and records the data regarding registration for vaccination of COVID-19 vaccines as a digital asset. Figure 3 presents the proof of delivery model.

### 3.3. Algorithms

**Figure 4:**
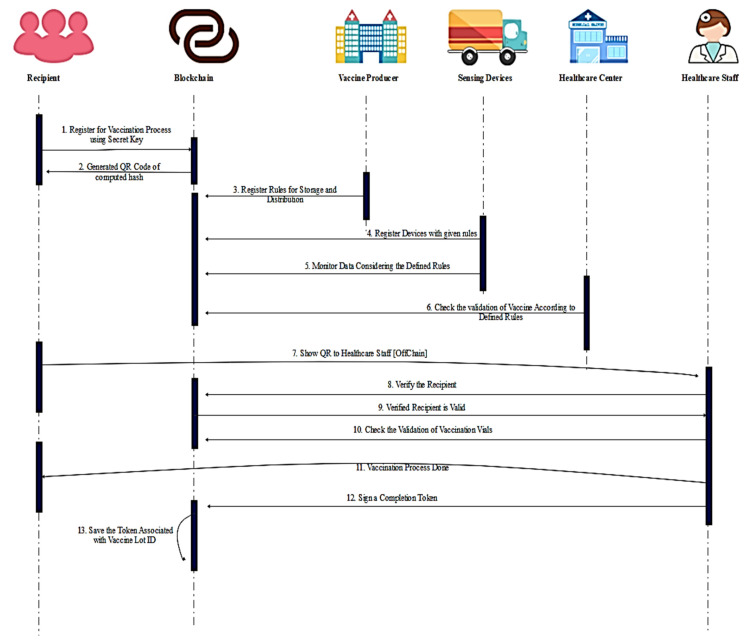
Sequence diagram of the proposed solution.

The proposed system has five participants in the network including the recipient, healthcare workers, laboratory staff, and administrative and healthcare center staff as shown in Figure 4. The detailed execution of recipient registration and chain monitoring of distribution process are described in the following subsections:

#### 3.3.1. Recipient Registration

The process of recipient registration in the blockchain network is shown in Algorithm 1. The objective of the registration process will help to verify the identification of the recipient. Firstly, the recipient will generate a key that will be secret and will be saved off chain in order to preserve the recipient’s data anonymity and privacy on the chain. This secret key (SK) will be used later to prove the identity of the recipient. A Markle proof [48] is used for the identification process; the root node (R) will contain the computed hash of recipient secret key and computed hash of recipient address (RA) as represented in Equation (1).

(1) $$R_{Hash}={\left(SK_{HASH},RA_{HASH}\right)}_{Hash}$$

$R_{Hash}$ is the root of the Markle tree that contains $SK_{HASH}$ and $RA_{HASH}$ will be stored on the blockchain network after the mining process, Once the transaction is stored onto the blockchain network a QR code of *R_Hash_* will be generated and that QR code will be used as an identification code by the vaccine recipient. During the vaccination process the healthcare workers will recompute the *R_Hash_* using the recipient’s secret key.

**Algorithm 1** Recipient_RegistrationV_ID: Vaccine Lot ID SK: Secrete Key C_HASH_: Computed Has hR_HASH_: Recipient’s Has hR_ADDRESS_: Recipient’s Address1.mapping (address V_ID = >int) public V_ID2.mapping (address recipient = >bool) public R_ADDRESS_3.mapping (byte R_HASH_ = >address) Recipient_Registeration4.Procedure Recipient_Registration (SK, R_HASH_, R_ADDSRESS_)5.C_HASH_ = (R_HASH_ + SK)_HASH_6.return C_HASH_7.end Procedure

#### 3.3.2. Chain Monitoring of the Distribution Process

The objective of this process is to make the distribution process of vaccines transparent with the help of IoT sensor devices. With the help of smart contracts, the sensor data is continuously evaluated and deployed on the blockchain network as shown in Algorithm 2. Initially the laboratory admin will set some parameters as rules for the storage and distribution of vaccine vials:

(2) $$R_{1}=T_{MIN}\;<\;T_{CAPTURED\;BY\;SENSORS}\;<\;T_{MAX}$$

(3) $$R_{2}=Time_{TAKEN\;BY\;DISTRIBUTOR}\;<\;Time_{LIMIT}$$

According to the given rules the freezing device will initiate and depending upon the distance between source and destination a time limit will be set. All the defined rules will be mapped by the vaccine lot identification (VL_ID). All the data captured by IoT sensors will be stored on to the blockchain network and the transaction will contain VL_ID, Rule 1, Rule 2, and the temperatures captured by the sensors.

**Algorithm 2** Vaccine_DistributionV_ID: Vaccination Lot ID F_Temp: Freezer Temperature F_Time: Freezer Start Time M_rules_: Monitoring Rules T_MIN_: Minimum Temperature T_MAX_: Maximum Temperature T_CAPTURED BY SENSOR_: Temperature Captured by the Sensors1.Mapping (byte V_ID = >int) public V_ID2.mapping(string rules = >rules) public M_rules_3.Mapping (address F_Temp = >V_ID) public F_Temp4.Mapping (address F_Temp = >M_rules_) public M_rules_5.Mapping (address F_Temp = >time) public time6.Mapping (bytes V_ID = >rule[]) public _monitored_values7.Procedure Vaccine_Distriution (V_ID, M_rules_)8.Set modifier = Vaccine Producer9.If (id == Vaccine Producer) then10.Access granted11.Set F_Temp to M_rules_ [Temp]12.Set F_Time to block.timestamp13.Store F_Temp to rule []14.Store F_Time to rule []15.Add (rule)16.End If17.Else18.Access denied19.End Else20.If (T_MIN_ < T_CAPTURED BY SENSOR_ < T_MAX_ && TIME_TAKEN BY DISTRIBUTOR_ < TIME_LIMIT_):21.Vaccine lot is valid22.Return the details of vaccine lot23.End If24.Else:25.Not valid26.Return Vaccine lot is not suitable27.End Else28.End Procedure

#### 3.3.3. Tampering Checking for Vaccine Vials

The responsibility of this process is to validate the transactions and check the block whether it is tamperproof or not. When any entity tries to tamper with the data, it goes through a process of checking where the hash will be compared by the previous block hash if they match than it is a valid block else that is an invalid block as shown in Algorithm 3.

**Algorithm 3** Tampering CheckV_ID: Vaccine Lot ID1.Procedure Tempering Check(Fake_ID_, block of the network, succeeding block)2.Fake_ID_ is the new V_ID3.Forged/Modified4.a = already stored block of the network5.a1 = succeeding block6.Tampering done by V_ID will be store in a7.If a1.previous_hash! = a.hash then8.Print (“tampered block not valid”)9.End If10.Else11.Print (“tampered free valid block”)12.End Else13.End Procedure

#### 3.3.4. Feedback for Received Vaccine

The recipients who have received their vaccination will fill out the feedback regarding the process starting from the registration till successfully being vaccinated, also about any post-vaccination symptoms or any side-effect encountered as given in Algorithm 4. The recipient will get the access only if the recipient identification in valid. After the validation process the recipient will submit the feedback and save the data on blockchain. In case of any post vaccine effect the peers can check the fault with the help of vaccination lot identification. The data is immutable and this feature disables any kind of tampering by third parties.

**Algorithm 4** Feedback for Received VaccineR_ID_: Recipient’s ID V_ID: Vaccine Lot ID1.Procedure Feedback(R_ID_)2.If R_ID_ is valid then3.Grant access4.mapping(bytes V_ID to String feedback) public feedback5.mapping(bytes V_ID to bool side_effect) public side_effect6.Save feedback to blockchain7.End if8.Else9.R_ID_ is not valid10.End else11.End procedure

Before starting the distribution process the vaccine producer has to provide the set of rules such as freezer temperature limits in order to initiate the process. The freezer time will automatically get initiated as and when the blockchain network transacts the first block that is called as genesis block of the blockchain network. As freezer time equals to the current time of the block.

**Figure 5:**
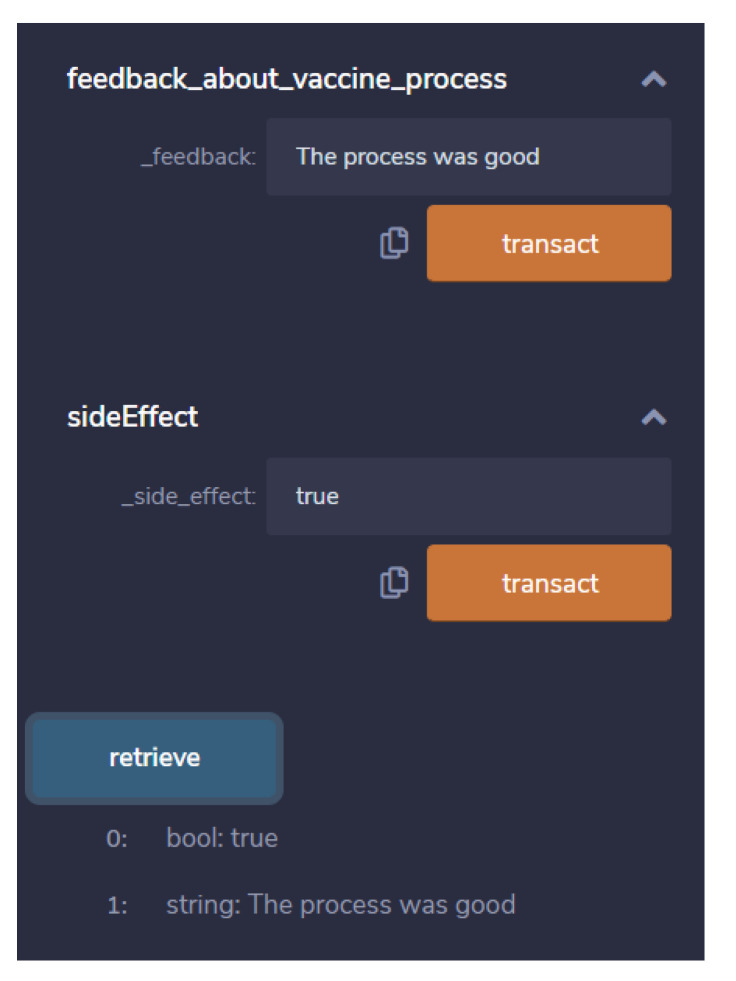
Snapshot of feedback and side effect contract execution.

The feedback page about the vaccination process. The first consists of feedback that is of string type the user can directly type the feedback. Secondly, side effect is of Boolean type it will only take either true or false values as shown in Figure 5. With the help of feedback feature the conflict in vaccination process are easy to identify and check. Also, it will be recorded in blockchain network for the future use to analyze the vaccination process.

## 4. Results

**Figure 6:**
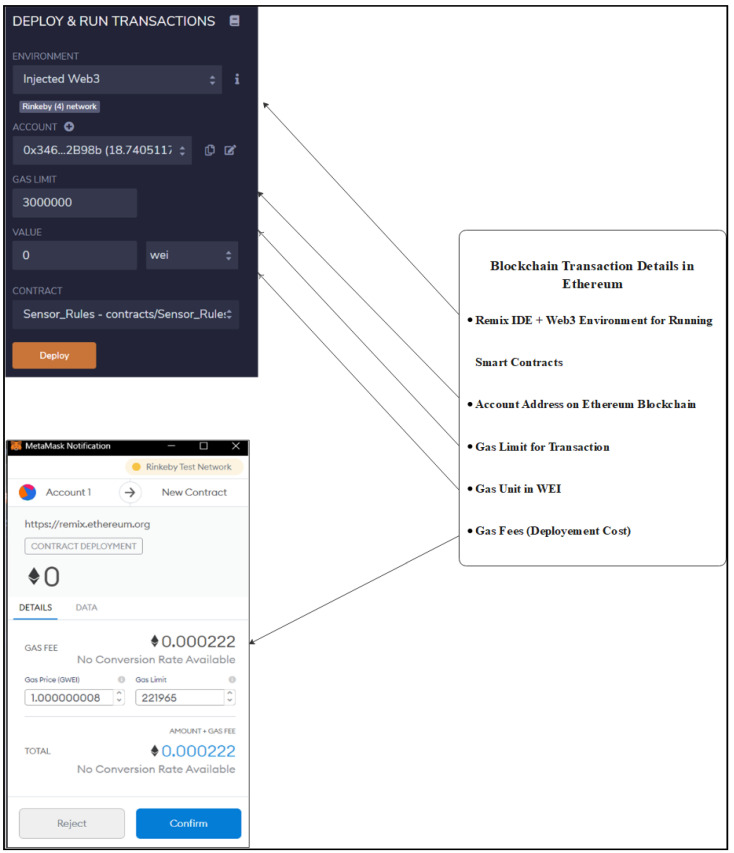
Metamask and Remix IDE.

In this section, the authors discuss the environmental setup, tools for simulation and outcomes of the proposed solution. Ethereum blockchain is used for the simulation process as it is an open-source platform. The Ethereum test network that is RinkeyBy is used for the deployment process of smart contracts [49]. Ethereum is a public blockchain that is used to develop decentralized applications (DApp) [50]. The Remix integrated development environment (IDE) used in order to assess the performance of the proposed solution [51]. Remix IDE provides a platform to write, execute and test a smart contract. The object oriented contract language (OOCL)-based Solidity language is used for the development of smart contracts [52]. MetaMask is used to connect Remix IDE with the Ethereum test network RinkeyBy; it works as a bridge between them as it is an extension in the browser as shown in Figure 6 [53].

### 4.1. Simulation Results

The specifications of the system that is used for the simulation are: 2.4 GHz CPU, 8 GB Random Access Memory (RAM), Intel core i5 CPU and a 2 TB HDD. The considered factors that are used to calculate the performance of the proposed solution are as follows:

#### 4.1.1. Gas Consumption for Verification and Deployment of Smart Contract

**Figure 7:**
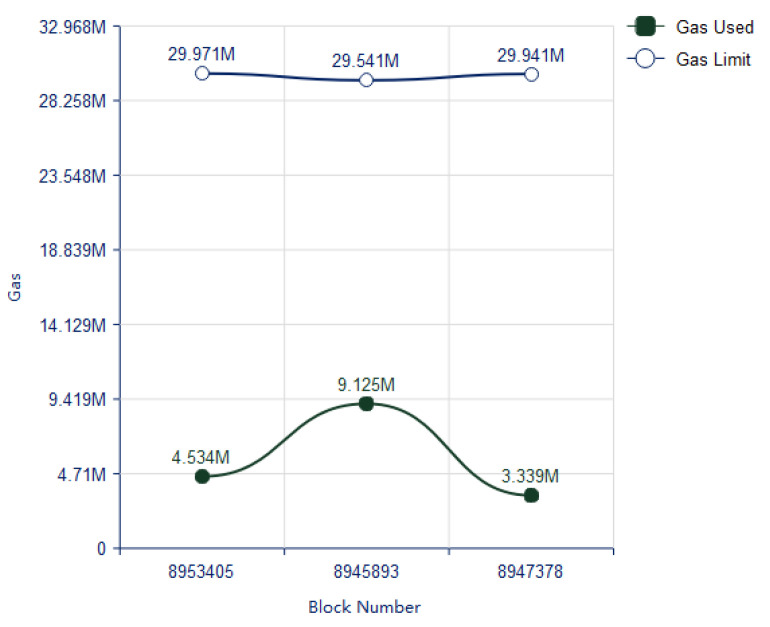
Gas limit and used gas for verification and deployment of smart contracts.

The evaluation of gas consumption for the verification and deployment of smart contract is done. In Figure 7 the maximum gas limits and the consumed gas is presented with respect to contract block number. However, the equations have been used for the evaluation of gas fees to ether coin and ether coin to US dollars are as follows:

(4) $$Cost_{Ether}=Gas_{Consumed}*\;0.007742$$

(5) $$Cost_{USD}=Cost_{Ether}*\;228.30$$

∴1 Ether=228.30USDollar

**Figure 8:**
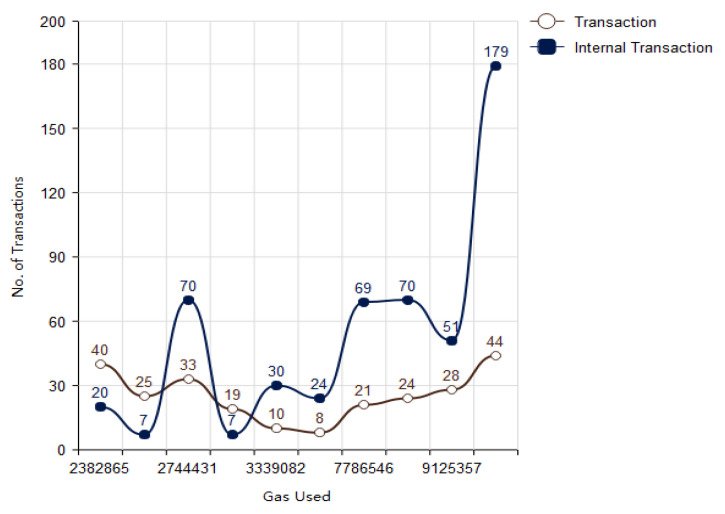
Gas used with respect to number of transactions.

In blockchain a single block consists of various internal and external transactions. In Figure 8 the relationship between transactions and gas consumed has been presented. The relation concludes as the number of transactions increases the gas consumption will also increase therefore, they are directly proportional to each other:

(6) No. of Transaction ∝Gas Consumption

**Figure 9:**
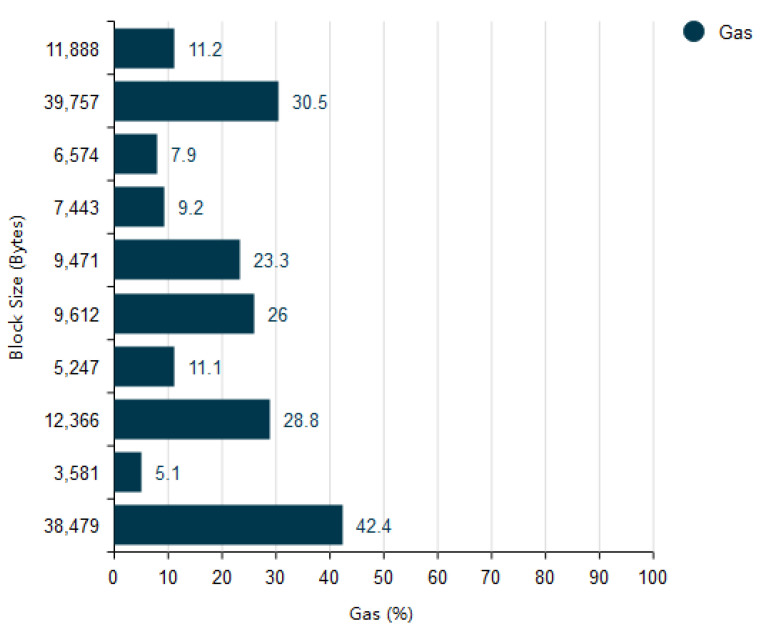
Gas percentage consumed according to block size.

In Figure 9 the relationship between the gas consumed based on the size of the block is shown. The relationship shows as the block size increases the gas consumption will also increase as shown in figure when the block size is 3581 the gas consumed percentage is 5.13% and when the block size is 38479 then the gas consumption percentage is 42.4% which shows both gas consumption and block size are directly proportional to each other.

(7) Block Size ∝ Gas Consumption

#### 4.1.2. Mining Time

**Figure 10:**
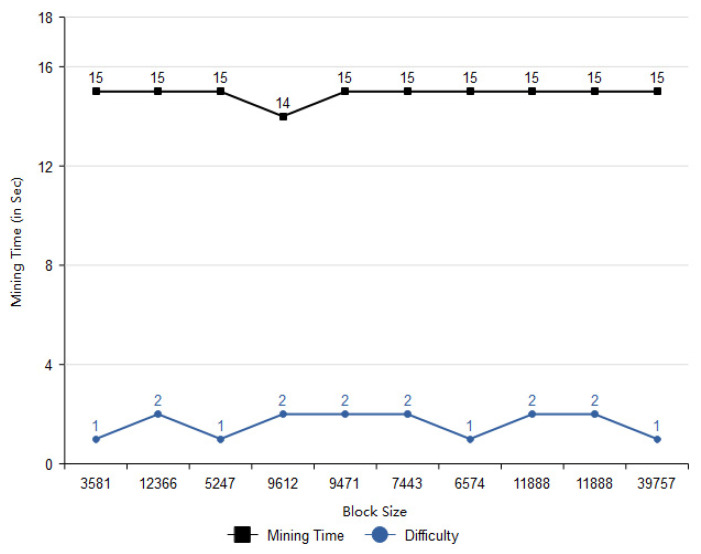
Mining time and difficulty with respect to block size.

In order to compute the mining time with respect to the particular block size, a bar graph is plotted as shown in Figure 10. The authors have provided different size of blocks in order to investigate the time of mining for each block. As shown in Figure 10, difficulty is a measure of unit to calculate the difficulty during the mining process. It has been observed that the time of mining does not depend upon the size of block. However, the mining time is dependent on the mining process of the network.

#### 4.1.3. Transaction Cost

**Figure 11:**
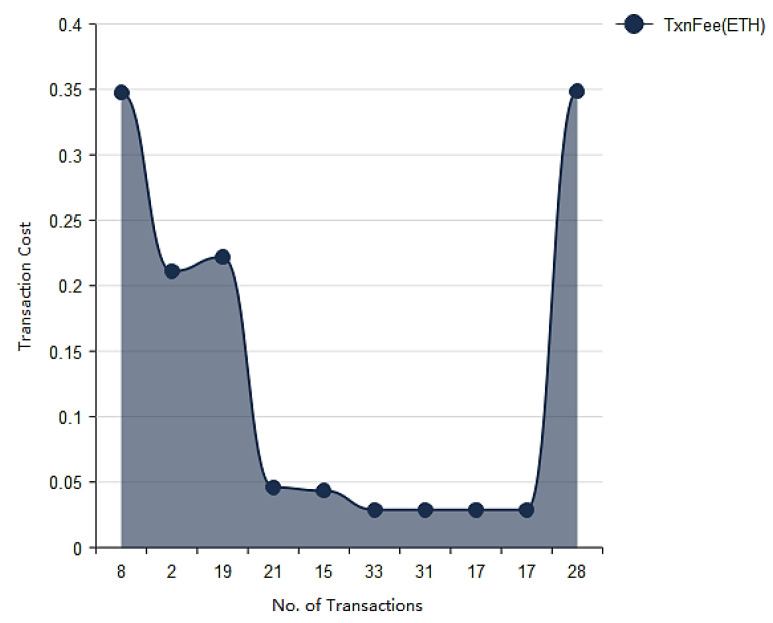
Transaction fees versus number of transactions.

**Figure 12:**
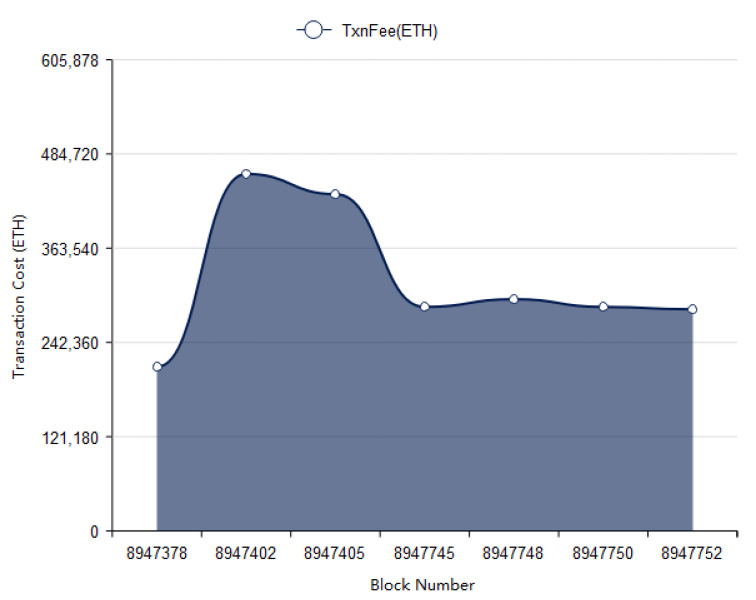
Block number vs transaction cost for distribution pages.

In Figure 11 the cost of each transaction has been presented depending upon the number of transactions done by respective block. As shown in figure on the x axis the transaction cost has been shown and on y axis block number (block ID) has been presented. The transaction cost depends on the type of input also on the number of transactions in a block. The transaction cost is in Ethers. The transaction cost may vary according to the size of blocks in blockchain as shown in Figure 12.

#### 4.1.4. Testing UI of Smart Contracts

**Figure 13:**
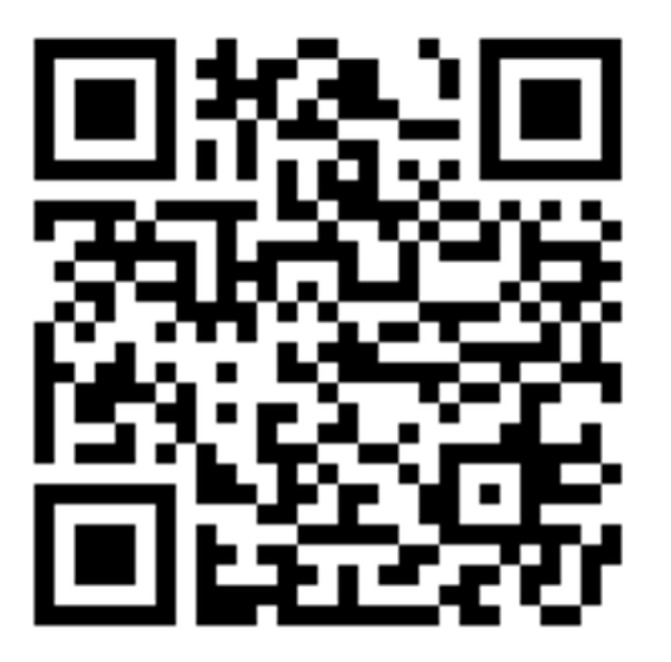
Recipient QR code.

The authors have tested the smart contracts on RinkeyBy network and performed the transactions by a decentralized application that is created using DApp Creator in order to perform the transactions on blockchain network. The recipient will provide a secrete key and then the QR code will be generated as shown in Figure 13. The QR code consists of computed hash by combining the secrete key with the recipient’s address using Equation (1).

#### 4.1.5. Discussion

A comparison of the earlier work has been described in Table 2 where the outcomes and scope of improvement of related work has been shown. To overcome the issues we have proposed the solution which enables various features such self-reporting, self-monitoring, immutable, and temper-proof, correctness, and transparency. Also the proposed solution provides patient data privacy as the private key is encrypted with a secret code provided by the user. Another feature is temper-proof when any entity tries to tamper the data, it goes through a process of checking where the hash will be compared by the previous block hash if they match than it is a valid block else that is an invalid block.

## 5. Conclusions and Future Scope

In this paper, a blockchain-enabled platform to increase the transparency during the supply and distribution of COVID-19 vaccination vials has been proposed. Smart contracts are developed to monitor the supply and distribution of vaccines. The proposed solutions offer features such as self-reporting, self-monitoring, immutable, and temper-proof, correctness, and transparency. During the distribution of COVID-19 vials, the producer will set some rules using smart contracts. Only registered recipients can get the vaccine after a verification process done by the healthcare worker. The simulation results of the proposed solution show the feasibility in terms of GAS computation and throughput of the transactions. After the analysis of obtained simulation results the consumption of gas, mining process, difficulty, transaction cost depends upon the input type or the block size for the deployment on blockchain. The solution provides: (i) a framework for data transparency, immutability, and efficiency of registration for COVID-19 vaccine campaign to avoid counterfeit and identification theft, (ii) a smart contract enabled framework for self-administering the vaccine distribution constraints in the cold chain about the fulfilment of COVID-19 vaccine, (iii) a framework for vaccine supply chain management that will enable the feature of tamper-proof, person identification and avoid counterfeiting.

In the future, an analysis of the collected data on feedback and data storage that can be done to identify the efficiency of vaccination vials also to check the suitable environment under which the vaccination vials can be stored. The proposed model stores all the transaction data in the blockchain, thus raising the concept of scalability and this issue still needs to be addressed.

## Figures and Tables

**Table 1:** Immunization COVID-19 vaccine and parameters ranges [13,14,15].

COVID-19 Vaccine	Frozen Vial	Thawed, Unpunched Vial	Thawed, Punched Vial
Pfizer	Frozen Temperature: −60 °C to −80 °C. Protection from sunlight. Preferred storage temperature: −70 °C.	Temperature: 2 °C to 8 °C and for up to 120 h or up to +25 °C for no more than 2 h.	Storage temperature: 2 °C to 25 °C. Avoid exposure to light during storage.
Moderna	Temperature: −15 °C to −25 °C Protection from light. Preferred storage temperature: −20 °C. Shelf life: 6 months.	Temperature: 2 °C to 8 °C, and for up to 12 h at 8 °C to 25 °C. Avoid exposure to sunlight during storage.	Storage temperature: 2 °C to 25 °C. Punched Vials should be protected from light.
Covisheild	Temperature: 2 °C to 8 °C. Do not freeze.	Storage temperature: 2 to 8 °C.	Stored temperature: 2 °C to 25 °C.
Covaxin	Temperature: +2 °C to +8 °C. Do not freeze.	Storage temperature: 2 °C to 8 °C.	Storage temperature: 2 to 8 °C.
Oxford AstraZeneca	NA	Storage temperature: 2 °C to 8 °C.	Storage temperature: 2 to 8 °C.

**Table 2:** Related work.

Ref.	Technology Used	Outcomes	Scope of Improvement
[24]	Agent-based modelling technique Simulation Tool	A drive- through simulation tool for vaccination is proposed to enhance the process of vaccination.	Lack of security No record of vaccine vial lot No solution for counterfeiting Patient’s data privacy
[29]	IoT Remote Monitoring	A solution for ensuring the transparency and increasing the coverage of vaccines in the remote regions using IoT sensor devices to track the carrier’s location, humidity and temperature.	Lack of security Counterfeiting is an issue Vaccine data is prone to tampering Patient’s data privacy
[33]	Blockchain Smart Contracts	The use cases related to technology like blockchain can help to manage the pandemic of COVID-19 by tracing of contacts, data sharing of patients, supply and distribution management are presented	Counterfeiting is an issue Vaccine data is prone to tampering Patient’s data privacy
[35]	Blockchain IoT Smart Contracts	A solution based on blockchain and IoT for privacy preserving of home quarantine patients.	Counterfeiting is an issue Vaccine data is prone to tampering No tracking data of COVID’19 vaccine
[36]	Blockchain	An incentive policy has been introduced for the betterment of government strategies for fighting the battle of coronavirus.	Lack of security Counterfeiting is an issue Vaccine data is prone to tampering Patient’s data privacy No tracking data of COVID’19 vaccine
[38]	IoT Blockchain	An integrated framework of blockchain and IoT for supply chain of pharmaceuticals where the counterfeiting prevention and temperature monitoring of drugs are of utmost importance.	Vaccine data is prone to tampering Patient’s data privacy No tracking data of COVID’19 vaccine
[39]	Machine Learning	A recommendation system using blockchain and machine learning for the management of the drugs supply chain.	Lack of security Counterfeiting is an issue Vaccine data is prone to tampering Patient’s data privacy No tracking data of COVID’19 vaccine
[40]	Blockchain	A transparent drug data flow using the Gcoin blockchain in order to eliminate the counterfeiting of drugs.	Lack of security Vaccine data is prone to tampering Patient’s data privacy No tracking data of COVID’19 vaccine

## Data Availability

Not applicable.
